# Supplementary material for: Development of the terminal air spaces in the gray short-tailed opossum (Monodelphis domestica)– 3D reconstruction by microcomputed tomography
Source: PLoS One. 2024 Feb 16;19(2):e0292482. doi: 10.1371/journal.pone.0292482 (PMC10871483; doi:10.1371/journal.pone.0292482)
Supplement: Attachment — Submitted filename: Comments to reviewers.docx [file pone.0292482.s001.docx]

Comments to Reviewer 1:

I thank the referee for his overall positive comments and the critical reading of the manuscript. I tried to address all points raised and revised the paper accordingly.

Major points

-P7 L8: The SEM pictures were included in the study to show structural details of the 3D architecture of the developing lung, which can be seen with a better resolution and image sharpness in SEM pictures than with the µCT. They were indeed used to make selected measurements of airspace diameter and septum thickness. However, I agree that a proper morphometric measurement should be applied to present reliable data. Therefore, I included morphometric measurements of airspace diameter and septum thickness of the lungs of the scanned µCT-specimens. Using the fractionator method, the µCT-scans of the lungs were digitally sectioned into eight parts, ensuring that the whole lung was sampled. Eight digital pictures were taken from the 2D sections of the lung at the same magnification (ensured by the same scale) for each animal and analysed using Image J software. The program was calibrated with the scale bar and a line was randomly cast over the image of the lung. On each digital photograph 5 airspace diameter and airspace septa intersecting with the line were measured, yielding a total of 40 for each lung. The values for single specimen and group means are presented as mean +- standard deviation in Table 1. A description of the morphometric measurement was included in the method section under 2.6.

For a better comparison of the SEM to the µCT specimens I added specifics and details (e.g., body weight, fixation, lung section) for the eight SEM specimens in Table 1. The former Table 1 was replaced by the former supplementary table. It now contains all details and results for the single specimens investigated and presents the group means ± standard deviation.

-P9 L15 For the reconstruction manual tracing on 16-bit pictures (16000 gray scale values) with a tolerance of 1000-1200 gray scale values around the first segmented gray value was used to ensure that the entire airspaces were captured. A paragraph has been added in 2.5.

-P9 L16 The segmentation process and creation of ROIs are explained in more detail under 2.5

The segmentation used a region grower tool, that marks all areas of the same density-value connected to each other to create a region of interest (ROI). The tissue density is mapped to gray values, so that tissues of the same density appear in the same gray scale value. A tolerance of 1000-1200 gray scale values around the first selected gray value of the ROI was given. New marked areas were included in this ROI. With that tool the entire bronchial tree was manually traced, beginning from the extrapulmonary main bronchi to the terminal bronchioles, and it was visually ensured that only air spaces were included. In that way pulmonary blood vessels and other air-filled areas in or between the lung segments were excluded from the segmentation. Calculations of volume and surface area are built in functions of Volume Graphics Studio Max. With segmentation a ROI will be created, which has a certain volume and surface area. The first ROI “bronchial tree” contained the entire bronchial tree of the lung (see Ferner and Mahlow, 2023). The surface area and volume of the ROI “bronchial tree” was calculated by the program. In a next step the ROI of the bronchial tree was copied and then extended to include the terminal air spaces. The resulting ROI “entire air spaces” included all conducting and terminal air spaces of the lung. Volume and surface area were determined for the ROI “entire air spaces”. By subtraction of the surface area and volume of the ROI “bronchial tree” from the ROI “entire air spaces”, the surface area (SA) and volume of the terminal air spaces (VA) resulted.

-P9 L18 As pointed out above the values VA provided in the results and table 1 are volumes of the terminal air spaces, since the volume of the ROI “bronchial tree” was subtracted from the ROI “entire air spaces”.

P9 The variables VL, VA and SA have been now introduced and explained in 2.5 of the methods section. I don`t no how exactly Volume Graphics calculates the surface areas and volumes, since it is an inbuilt function of the program, but I believe these are just mathematical calculations of the segmented volumes.

-P10 Morphometric measurements have been conducted to estimate septum thickness and air space diameter. The fractionator method has been applied to ensure that the whole lung was sampled and randomness in the measurement was introduced, as I already have explained in the first point of the reply to the reviewer. A new section (2.6) was included in the methods to explain the morphometry. The mean and standard deviation for the single specimens and age groups as well are given in table 1.

Roughness of µCT volume renderings

Segmentation was carried out with a region grower tool (with a tolerance of 100-1200 gray scale values around the first selected gray value of the ROI). However, since tracing was performed manually it is possible that not all surfaces, especially in the later postnatal stages, might be reproduced perfectly. This leads to an artificial roughness in some segmentations. I agree this might influence the calculations of surface areas and volumes of air spaces. In these cases, the surface area might be overestimated and the volume of terminal airspaces would be lower than in reality. I added a short discussion of this issue in the method section.

Minor points

-“air spaces” are used consistently in the text now. The sentence P12, L10-11 has been deleted.

- P7 L17 “first weeks of life” are replaces by “postnatal period”

- P7 L1 The term “adult” has been defined now. For the study primi- or multiparous females approximately one year old were used. Since the young Monodelphis (0-14 days) are firmly attached to the maternal teat (removal would be very painful and cause injuries), the mother had to be euthanized to obtain these stages. For efficiency, the mothers were used for lung fixation and dissection.

- P10 L5 A sentence at the beginning of the results section clarifies that all values for volume and surface area are group means of the respective age group.

-P15 L25 The part of the discussion has been revised to be consistent in present tense.

- P17 L16 and L24 The values have been changed from µl to mm³ to provide better comparability.

-P30 Table1 V_L_, V_A_ and S_A_ have been adjusted to meet the rest of the manuscript.

P30 Table1 The values are now given as means and standard deviation, for single specimens and age group.

Comments to Reviewer 2:

I thank the reviewer for his thoroughly reading of this manuscript and the comments and recommendations. I tried my best to amend the paper according to the reviewer suggestions.

Major points

I followed the reviewer’s suggestion and modified figure 11. It now shows the individual data points for lung and air space volume and surface area of *Monodelphis domestica* together with morphometric literature data of marsupials and eutherians (data from rat, cattle, pig and sheep a merged to the eutherian data set) to make the acquired data better comparable.

Presentation of the data: I followed the reviewer’s suggestion and modified the presentation of all data to mean +- standard deviation. Table 1 includes now all values of single specimens and group means. The supplementary table is not necessary anymore and will be omitted.

A sentence to the analysis and plotting of the data in Figure 11 has been added in the method section 2.5 after explaining how VL, VA, SA were obtained.

Colorful segmentations of the functional lung units: A paragraph at the end of 2.5 explains how the colored segmentations of the functional lung units were obtained. The color can be individually chosen for the segmented ROIs (“set interval color”). To set the neighbouring airspaces apart from each other different colors were chosen.

“Development curve of lung lobe volumes”: I agree that this information would be interesting and it might be possibly to generate the information from the entire segmented lungs. For this the ROI of the entire air spaces must be copied and all the parts of the lung deleted that do not belong to the lung lobe of interest. This might be easily done in early postnatal stages since the air spaces are large and well set apart from each other. In later stages the terminal airspaces of the single lung lobes must be confirmed by the course of the lobar and terminal bronchioles. In my opinion for this paper this is going too far. Besides I am not able to do any segmentations at our CT-Lab at the moment, since all computers are shut down after a cyber attack with no time frame given when work will be resumed. But maybe I will follow the reviewer’s suggestion and investigate it as a follow-up project. For the development of the bronchial tree information is available (see Ferner and Mahlow 2023). Not in terms of volumes, but in number of airway branches (Fig. 13) and airway generation (supplementary figure). The results show that the caudal lobes have a higher increase in airway generation and especially in total number of airway branches compared to the other lung lobes.

The given DOI-link for the published data does not work due to continuing restrictions to access the data at the Museum für Naturkunde resulting from the Cyber-attack from mid-October. Since I can not estimate when the link will be working again, I created new Data-Doi’s with figshare (Data: https://doi.org/10.6084/m9.figshare.24764187; Original images: https://doi.org/10.6084/m9.figshare.24763497; 3D-images: https://doi.org/10.6084/m9.figshare.24771213 and videos: https://doi.org/10.6084/m9.figshare.24764397) to make the data publicly available.

Minor points

I followed all reviewer’s suggestion and amended the text accordingly.

-Data availability statement:

I created a Data-DOI (https://doi.org/10.7479/cy7h-j182) containing all original data, images and 3D-animations of the reconstructed terminal airways. Unfortunately, this link is not working at the moment, since the Museum fuer Naturkunde is still suffering from a Cyber-attack that took place mid-october. Since there no official time-line is given when things will run properly again, I decided to create a new Data-DOI (Fig-Share) to provide the data asap.

Detailed Comments:

I followed the reviewer’s suggestion for Abstract, key words and Introduction and amended the text accordingly.

Introduction:

P3, L11, L15: I changed the text accordingly.

P3, L20: The citation Modepalli et al. 2018 has been added.

P4, L7, 12: I changed the text accordingly.

P4, L12: “Mice” and the citation Mund et al. 2008 have been added

P5, L26: “3D reconstruction” replaced “3D remodeling”.

P6, L4, L10: I changed the text accordingly.

P6, L26: The location of the MfN has been added.

P7, L4: has been done.

P7, L6: The age stage adult has been defined now. Adult animals were generally primi- or multiparous females one year old.

P7, L7: I changed the text accordingly.

P7, L9 and 22: Two sentences clarify why TEM and SEM samples were included in the study. “The TEM samples were used for further ultrastructural analysis, which is not subject to this study”. “The (SEM) samples were viewed and photographed in a scanning electron microscopic (LEO 1450 VP, Carl Zeiss NT GmbH) to see ultrastructural details of the 3D architecture of the lung.”

P7, L17: I specified the fixation times in the text, generally 1-2 days for Bouin and for weeks/months in Karnovsky until µCT scans were performed.

P7, L18: The comma has been inserted.

P7, L27: “The TEM samples were used for further ultrastructural analysis, which is not subject to this study.” This has been stated in the paragraph. See also comments to reviever 1.

P8, L5: "µCT imaging” has replaced “µCT”.

P8, L7: A Paragraph explaining the necessity for staining for µCT-scans has been added at the beginning of 2.3. “Comparative, functional, and developmental studies of animal morphology require accurate visualization of three-dimensional structures, but few widely applicable methods exist for non-destructive whole-volume imaging of animal tissues. µCT imaging in comparative morphology has been used in paleontology, where mineralized tissue, e.g., bones, were scanned. However, µCT-imaging of soft-tissue structures has been limited by the low intrinsic x-ray contrast of non-mineralized tissues. With sufficient contrast imparted to soft tissues, internal soft tissues, such as lung, liver, kidney, heart, intestine, skin and brain, can be made visible with µCT- techniques. With very simple contrast staining µCT imaging produces quantitative, high-resolution, high-contrast volume images of lung tissue. This is possible without destroying the specimens and with possibilities of combining with other preparation and imaging methods (histology or TEM).

Metscher et al. (2009) summarizes several simple and versatile staining methods for µCT-imaging of animal soft tissues, along with advice on tissue fixation and sample preparation. Based on this information, different staining protocols using inorganic iodine and phosphotungstic acid (PTA), were developed, tested and used to produce high-contrast x-ray images of the lung at different age stages (Table 1).”

P8, L13: In my opinion the reviewer’s suggestion to extend this paragraph leads too far. There were no reportable results obtained, the comparison in respect of shrinking resulted in comparison of volumes of PTA or Iodid stained animals (see table 1). We did not perform a scientific study on this subject.

P8, L16: I changed the text accordingly.

P8, L21: It has been changed according the reviewer’s suggestion.

P8, L26: The different kV, µA and projection-settings can be not obtained at the moment, since I do not have access to the VG data due to the shutdown after the cyber-attack at MfN.

P9, L13 Nexus was not used in this study. The sentence has been deleted.

P9, datasets: I can’t give you exact information about the pixel size of the acquired data sets right now, since I cannot open VG files. But the scans are based on a vector image of 1400 x 1480 pixel. The size of the acquired scan-files on disk depends on the size of the scanned sample (between 6 and 15 GB). For example, a small Araldite-block-scan of the whole upper part of a Neonate (e.g., Neonate 1965_1) generates a VG file of 10.6 GB. A whole neonate (2350_7), scanned in liquid results in a VG file of 12.5 GB. An adult lung (2117) resulted in a VG file of 15.1 GB.

The 3D-images I used for creating the plates Fig. 4,7 and 9 were saved as JPG-files, in a size range of 180-313 KB (Neonate 2350_7) to 203-282KB (Adult 2117). A 3D-rendering of the reconstruction (e.g. neonate video 2350_7; video turning bronchial tree and terminal airspaces) of 30 sec has a size of 52 MB.

P9; L15: The section title reads now: “Segmentation, visualisation and data analysis for 3D reconstruction”

P9, L12: The sentence in question has been deleted. Instead, two sentences explain how the segmentation was performed: “The segmentation used a region grower tool, that marks all areas of the same density-value connected to each other to create a region of interest (ROI). The tissue density is mapped to gray values, so that tissues of the same density appear in the same gray scale value.”

P9, L17: The paragraph has been deleted.

P9, L19 “Volume Graphics Studio Max” has been spelled out. The volume and surface area calculations are a built-in function of VG. With segmentation a ROI will be created, which has a certain volume and surface area. I can’t say, how VG exactly calculates the values. A paragraph has been added in 2.5.

P9, L20: The part of the sentence has been deleted.

P9, L21: I followed the reviewer’s suggestion fully.

P10, L9: The phrase has been deleted.

P10, L17: The phrase has been deleted.

P10, L20: The phrase has been deleted.

P10, L22: The sentence has been rephrased and the subjective statement has been deleted.

P11, L2: I changed the text accordingly.

P11, L6: I changed the text accordingly. The “architectural” complexity has been replaced by “structural” complexity and refers to Fig. 6H.

P11, L9: I changed the text accordingly.

P11, L14: The sentence has been deleted.

P12, L13: I changed the text accordingly.

P13, L1: I changed the text accordingly.

P13, L4: The surface area was calculated by VG Studio Max, I don’t know the exact algorithm of this.

P14, L11: normally the common species names are written lower case, but for the Gray short-tailed opossum both upper- and lower-case spelling is used. However, I decided to make it consistent and changed the species name to lower case.

P14, L5: I changed the text accordingly.

P14, L13: I changed the text accordingly.

P17, L4: I changed the text accordingly.

P19, L8, L9: I rephrased the sentence to homogenize the cited data.

P19, L15: I agree with the reviewer, that the lung volumes might tend to be lower than in functional lung (as already discussed in the manuscript), but due to differences in inhalation/exhalation status at the time of death variability in lung volume might result in any case. Therefore, I didn’t change the sentence.

P30: I followed the reviewer’s suggestion and table 1 shows now all values and the supplementary table has been omitted. Mean and standard deviation have replaced median and range.

Figure legends: All scale bars have been labeled in all figures and all statements about scale bars were deleted from the legends, since the scale bars are self-explanatory in the figures.

P31, P33: I followed the reviewer’s suggestions.
