# Supplementary material for: Development of the terminal air spaces in the gray short-tailed opossum (Monodelphis domestica)– 3D reconstruction by microcomputed tomography
Source: PLoS One. 2024 Feb 16;19(2):e0292482. doi: 10.1371/journal.pone.0292482 (PMC10871483; doi:10.1371/journal.pone.0292482)
Supplement: Attachment — Submitted filename: Comments to reviewers.docx [file pone.0292482.s002.docx]

Comments to Reviewer 1:

I thank the referee for his thoroughly reading of the manuscript and the helpful comments. I tried to address all points raised and revised the paper accordingly.

Major issues:
P11 L20f I do not completely understand the segmentation process, as both manual tracing and a region grower tool with a gray scale tolerance are mentioned. I conclude from that, that you did not manually trace the outline of every air space in every 2D image of your stack, but instead traced something like e.g. the center line (please specify) of the airways and extended that marking by region growing to the airway walls. Please elaborate what exactly was manually marked.

The reviewer is right. I did not draw the lines of the outline of the air spaces manually. Instead, I used the region grower tool to trace the walls of airways and air spaces. Starting from the centerline of the trachea (starting gray value), the region grower tool was extended to the tracheal wall. From there the ROI was extended by scrolling through the image stack and applying region growing to the airway walls and subsequently to the terminal air space walls. I included this sentence in the paragraph on page 12 for clarification.

P13 L16f The choosing of a random starting point is omitted in the description, but should be mentioned as it is an elementary part of the uniform random sampling scheme employed here. It might be added to the discussion, that the fractionator principle is employed here in a modified form.

I followed the reviewer’s suggestion. And amended the text accordingly. A sentence addresses the issues raised: “This requirement is met by choosing a random starting point and employing uniform random sampling using the fractionator principal in a modified form.”

P11 L20f 16-bit images contain 2^16 = 65,536 gray scale values, not 16,000

I followed the suggestion of reviewer 2 and deleted the gray scale values.

P12 L23f Please add a source for the literature data you refer to here

I added five references.

P25 L1 2.122 cm² is not 26 times 0.028 cm². The factor should be around 76, or one of the surface size values is off.

I thank the reviewer for the comment. I corrected the factor.

Minor issues:
P6 L5f X-ray *micro* computed tomography

I replaced “X-ray” by “micro-computed” throughout the manuscript

P6 L21 The abbreviation µCT is introduced here a second time

I deleted this part.

Table 1 The SD for 13 dpc is missing the “±”

the “±” by 13 dpc has been added.

Table 1 SEM specimen data could be moved to its own table (and might be referenced in the text on P9 L1)

I followed the reviewer’s suggestion and moved the SEM data to an own table (table2).

Table 1 PTA = *phospho*tungstic acid

PTA has been spelled out.

P8 L14 “instillation” instead of “installation”?

The correction has been done.
P9 L25 The abbreviation PTA is introduced here a second time

“phosphor tungstic acid” has been deleted, it is only PTA now.

P10 L19 No need for the “|” here

the “|” has been deleted.

P10 L18-24 The sentence stretches over seven lines. It might be broken up for increased readability

I broke the long sentence into 3 sentences.

P10 L21-26 The format *number* *unit* is used here, while it is *number* *space* *unit* everywhere else

I have added the spaces between number and unit.

P11 L13 One time *number* *unit* is used and right afterwards *number* *space* *unit*

The space has been added.

P13 L1f The figure should be referenced here

I referenced the former figure 11 here following the reviewer’s suggestion. It changed the order of the figures which had to be changed in the manuscript. Figure 11 became Figure 3, and all subsequent figures shifted to a number higher.

P13 L10 A fragment of a deleted section is left over here

The fragment has been deleted.

P13 L13 Inconsistent writing of air space/airspace

“air space” has replaced “airspace”
P13 L23 “µct” is used instead of “µCT”

“µct” has been corrected to “µCT”
P14 L2 “airspace” is used instead of “air space”

“air space” has replaced “airspace”

P16 L16 The word “staget” might be a typo of “stage”

The typo has been corrected.

P16 L21 “14dpn” is used instead of “14 dpn”

The space by 14 dpn has been added.

P16 L22 “Fig 8K” is used instead of “Fig 8 K”

The space has been added in “8 K”

P16 L24 Use of postnatal days instead of the established abbreviation “dpn”
P17 L10 Use of postnatal days instead of the established abbreviation “dpn”
P22 L13 Use of postnatal days instead of the established abbreviation “dpn” P25 L21 Use of postnatal days instead of the established abbreviation “dpn”
I use “dpn” now for consistency

P17 L12 “8K” the “8” is not needed here, I think

The “8” has been deleted.
P24 L9 The abbreviation VL has already been introduced in the segmentation section. No need to define it again

Lung volume has been deleted, It’s just V_L_ now.

P24 L9 For consistency: “2,629.33 mm³” could be used instead of “2629.33 mm³”

It has been done.

P24 L15f The bodyweights for the other marsupial neonates might be added to aid comparison

I have added body weights for the marsupial neonates.

P24 L23f Introduction of VL, VA and SA is not necessary here, as they have been defined before

(VA), (VL), (SA) has been deleted.

P25 L15 28 *dpn* for consistency

I added “dpn” for consistency.

P26 L3 Missing space between “35” and “dpn”

I added the space.

Comments to Reviewer 2:

I thank referee 2 for his in-depth review and the comments. I tried to address all points raised and revised the paper accordingly.

Table 1: Animals are sometimes listed with a space between the day and 'dpn' and sometimes not. It should be homogenized to being shown *with* a space as it is throughout the manuscript.
Line breaks in the table header should be corrected, but this is probably done in production at PLOS ONE.

The spaces have been inserted.

p. 8, l. 8: A space is missing between 2 and g.

The space has been inserted.

p. 10, l. 5ff: "The specimens had to be fixed...". I would write that the specimenst "were" fixed in the container with cotton balls to avoid movement artefacts during the scan and completely remove the sharpness part of the sentence. The 'sharpness' of tomographic data is decreased with movement artefacts, but also other factors.

I followed the reviewer’s suggestion.

p. 10, l. 18ff: Several instances where a space between number and unit are missing (ms and kV).

The spaces have been inserted.

p. 11, l. 13: "4dpn" is missing a space.

The space has been inserted.

p. 11, l. 20: [....] carried out on 16-bit images [...] I would remove "(16000 gray scale values)" as 16 bit images can record up to 65536 gray values, not only 16000.

I followed the reviewer’s suggestion and deleted the gray scale values.

p. 13, l. 17: The mentioned "Fractionator method" could be explained with a citation.

I inserted a citation for the Fractionator method.

p. 13, l. 25: "(Image J software; National Institutes of Health, USA)." should be cited according to [https://imagej.net/contribute/citing](https://deref-web.de/mail/client/OHGpVZYR6jc/dereferrer/?redirectUrl=https%3A%2F%2Fimagej.net%2Fcontribute%2Fciting) and the version used (either Fiji, ImageJ. Also, the software is titled without space between Image and J (also correct in abstract)

I added a citation for the original imageJ version I used for my measurements. All following

references had to be switched to a higher number than.

p. 16, l. 21: "14dpn" is missing a space.

The space has been inserted.

p. 26, l. 3: "35dpn" is missing a space.

The space has been inserted.

p. 19, l. 1: "...in thickness (12 μm) In the..." is missing a full stop after (12 μm).

I inserted the full stop.

Fig. 1-10: Thanks for inlcuding labeled scale bars in all these figures; this greatly helps with readability.
The scalebar of Panel O of Fig. 8 could have a black background behind the text :)

I gave a black background behind the scale and text.

Fig 11: The figure is greatly improved, but rather pixelated, even when downloading the original image from the EditorialManager. Its export quality should be increased for publication.

I fully agree with the reviewer and have already prepared a figure with higher quality to include in the final submission.
